# Supplementary material for: Most Monogenic Disorders Are Caused by Mutations Altering Protein Folding Free Energy
Source: Int J Mol Sci. 2024 Feb 6;25(4):1963. doi: 10.3390/ijms25041963 (PMC10888012; doi:10.3390/ijms25041963)
Supplement: Supplementary file 1 [file ijms-25-01963-s001.zip › ijms-2825014-supplementary.pdf]

Most monogenic disorders are caused by mutations altering protein folding free energy

Preeti Pandey[1] and Emil Alexov[1,*]

Department of Physics and Astronomy, Clemson University, Clemson, SC 29634, USA
* Correspondence: Corresponding author email: ealexov@clemson.edu

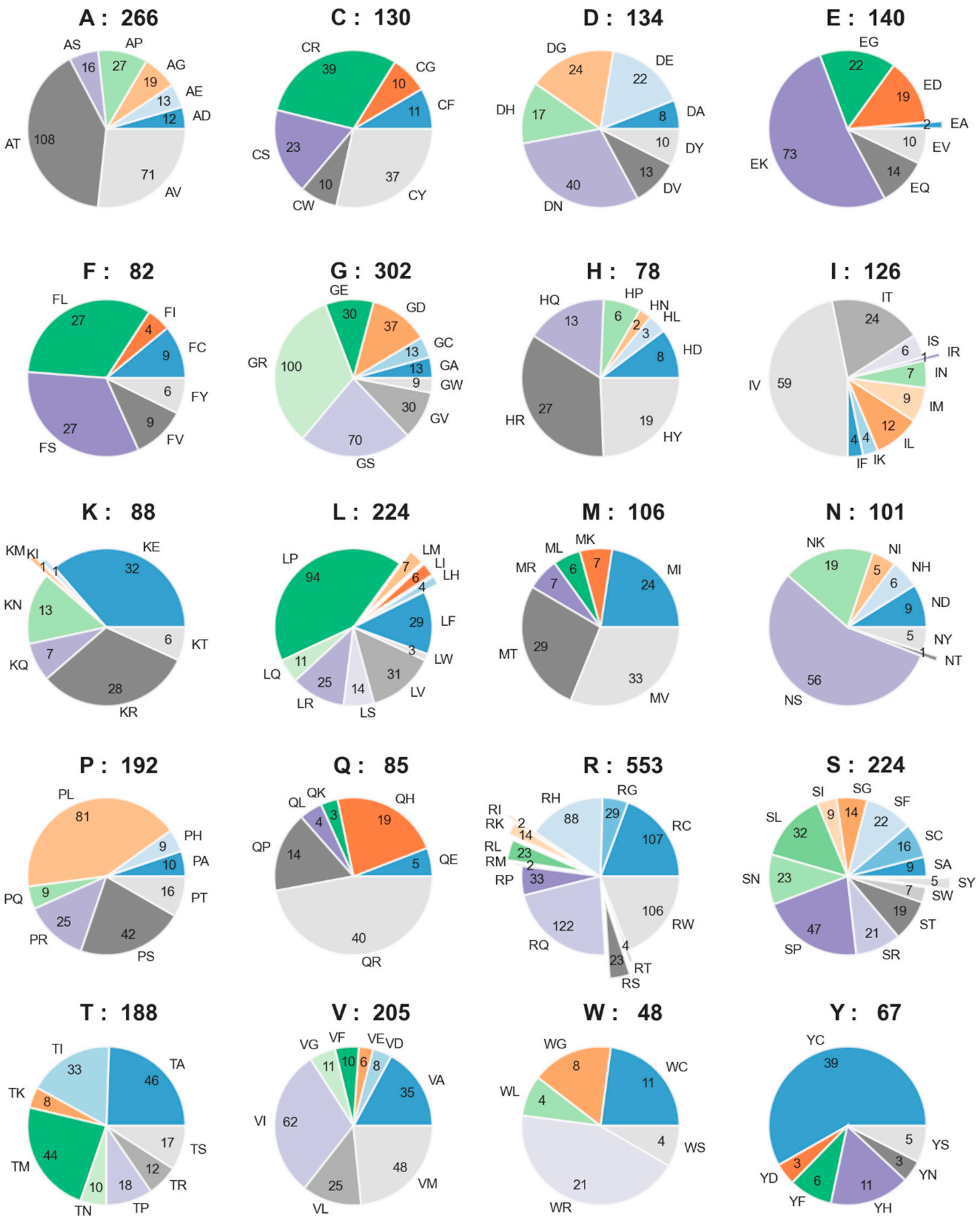

A : 266
AS 16
AP 27
AG 19
AE 13
AD 12
AT 108
AV 71
C : 130
CR 39
CG 10
CF 11
CS 23
CW 10
CY 37
D : 134
DG 24
DE 22
DA 8
DH 17
DY 10
DV 13
DN 40
E : 140
EG 22
ED 19
EA 2
EV 10
EQ 14
EK 73
F : 82
FL 27
FI 4
FC 9
FY 6
FV 9
FS 27
G : 302
GE 30
GD 37
GC 13
GA 13
GW 9
GV 30
GS 70
GR 100
H : 78
HQ 13
HP 6
HN 2
HL 3
HD 8
HY 19
HR 27
I : 126
IT 24
IS 6
IR 1
IN 7
IM 9
IL 12
IK 4
IF 4
IV 59
K : 88
KM
KI 1
1
KE 32
KN 13
KT 6
KQ 7
KR 28
L : 224
LP 94
LM 7
LI 6
LH 4
LF 29
LW 3
LV 31
LS 14
LR 25
LQ 11
M : 106
MK 7
ML 6
MR 7
MI 24
MT 29
MV 33
N : 101
NK 19
NI 5
NH 6
ND 9
NY 5
NT 1
NS 56
P : 192
PL 81
PH 9
PA 10
PT 16
PS 42
PR 25
PQ 9
Q : 85
QK 3
QL 4
QH 19
QP 14
QE 5
QR 40
R : 553
RH 88
RG 29
RC 107
RI 2
RK 14
RL 23
RM 2
RP 33
RQ 122
RW 106
RT 4
RS 23
S : 224
SI 9
SG 14
SF 22
SL 32
SC 16
SA 9
SY 5
SW 7
SN 23
ST 19
SP 47
SR 21
T : 188
TI 33
TA 46
TK 8
TM 44
TS 17
TR 12
TN 10
TP 18
V : 205
VG 11
VF 10
VE 6
VD 8
VA 35
VI 62
VM 48
VL 25
W : 48
WG 8
WC 11
WL 4
WS 4
WR 21
Y : 67
YC 39
YS 5
YN 3
YD 3
YF 6
YH 11

(a)

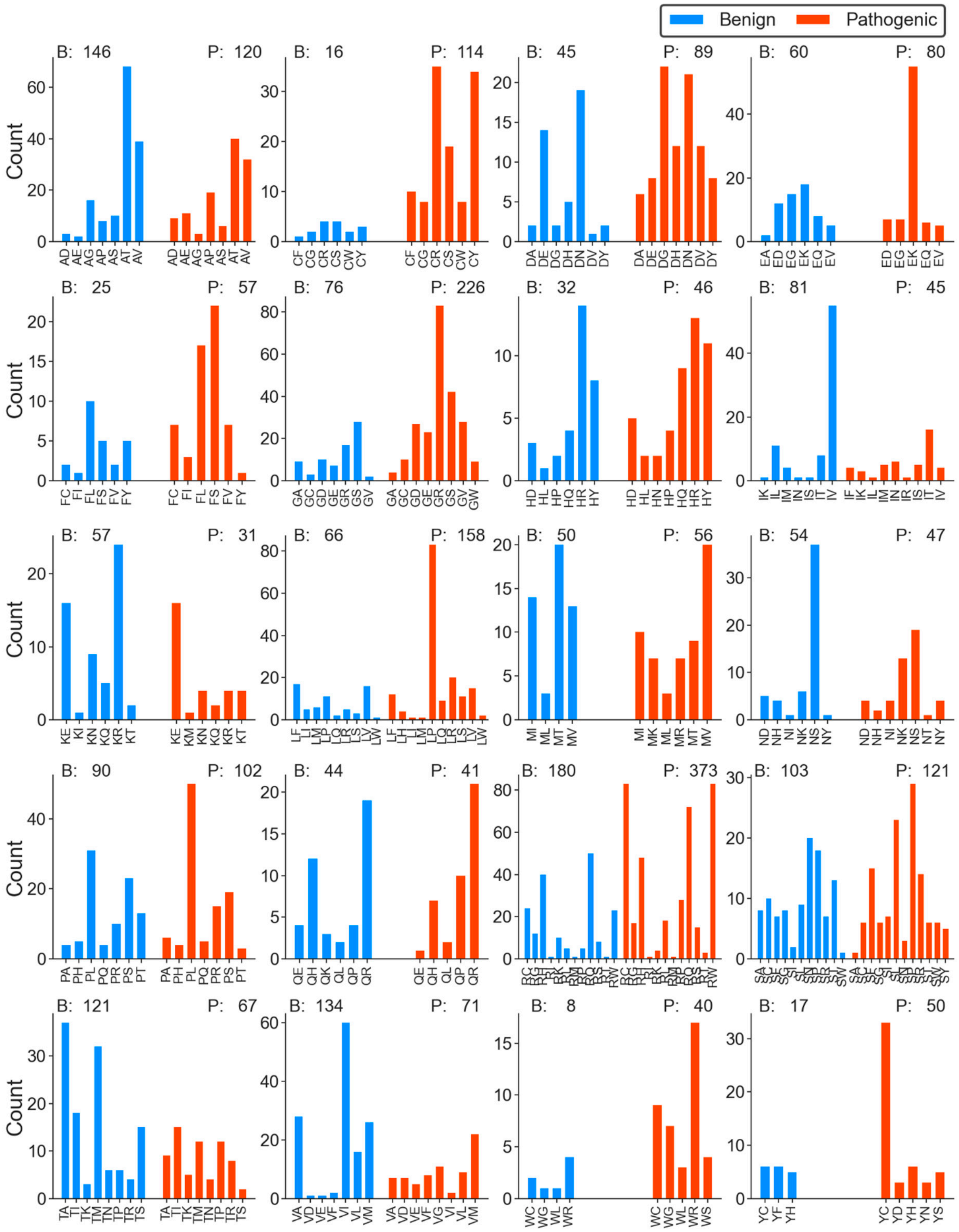

(b)

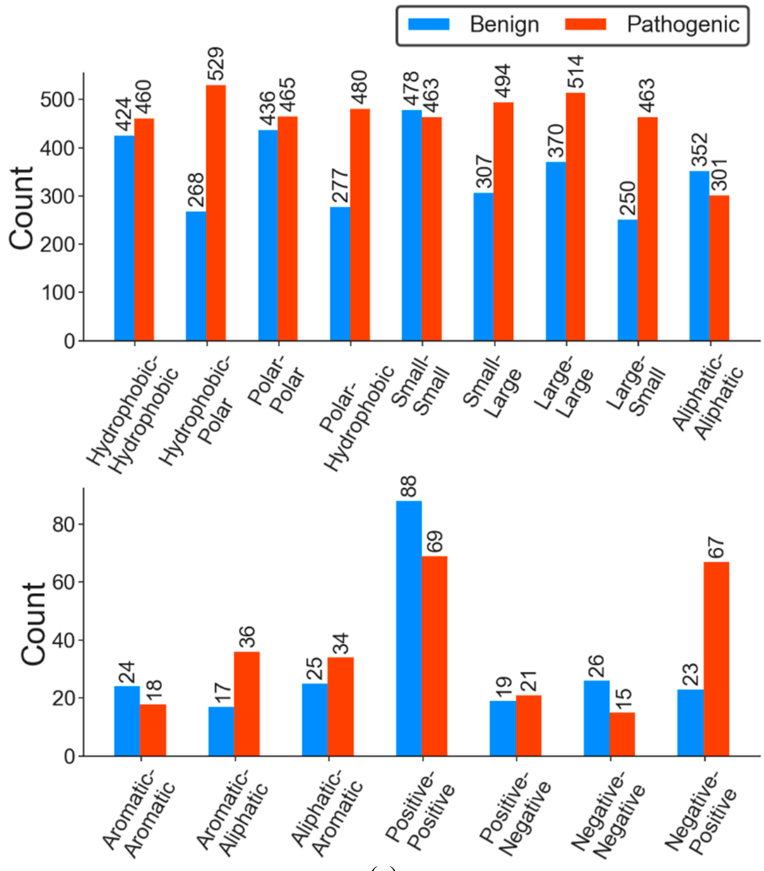
Benign
Pathogenic
Count
500
400
300
200
100
0
424
460
268
529
436
465
277
480
478
463
307
494
370
514
250
463
352
301
Hydrophobic-Hydrophobic
Hydrophobic-Polar
Polar-Polar
Polar-Hydrophobic
Small-Small
Small-Large
Large-Large
Large-Small
Aliphatic-Aliphatic
Count
80
60
40
20
0
24
18
17
36
25
34
88
69
19
21
26
15
23
67
Aromatic-Aromatic
Aromatic-Aliphatic
Aliphatic-Aromatic
Positive-Positive
Positive-Negative
Negative-Negative
Negative-Positive

(c)

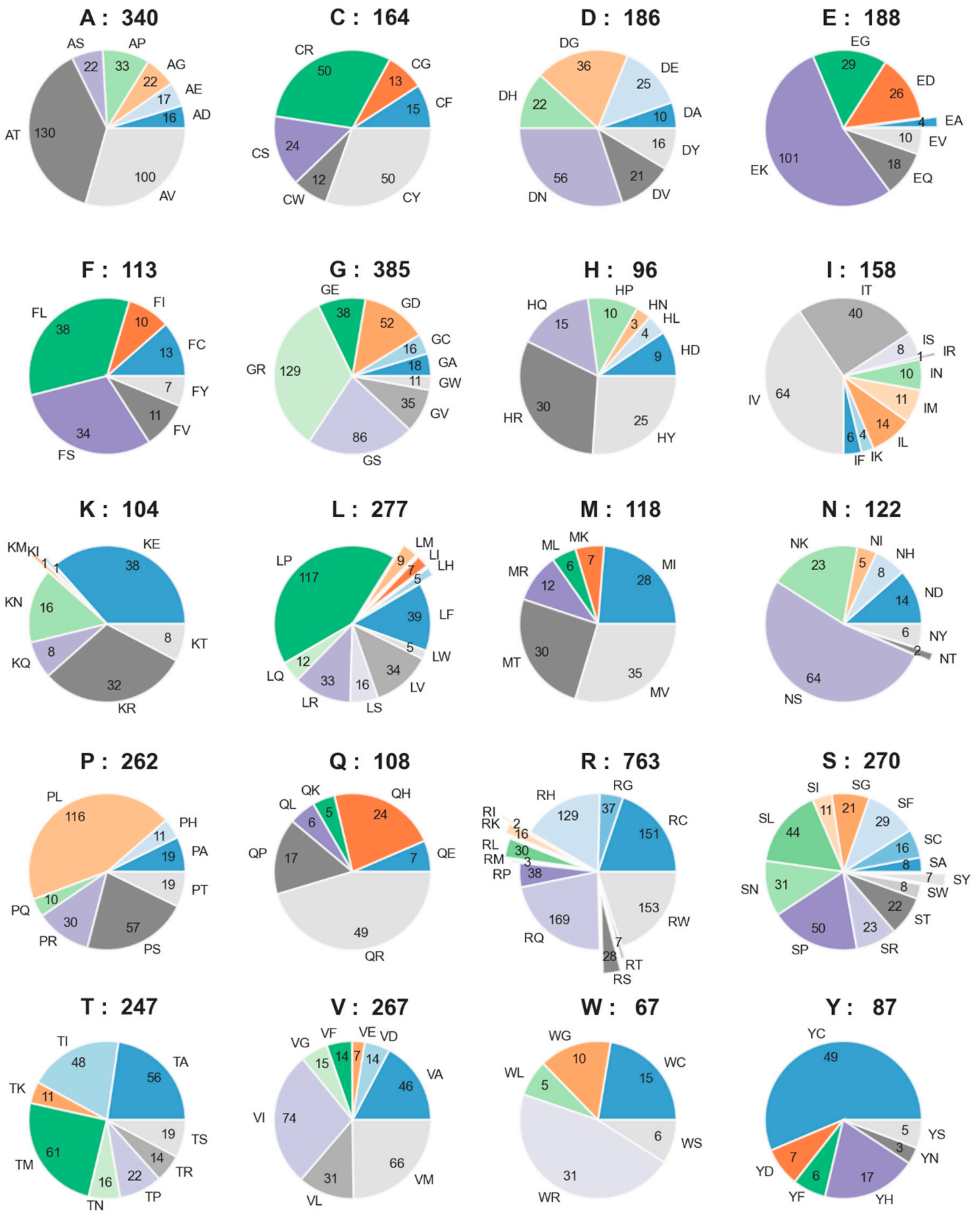
A : 340
AS 22
AP 33
AG 22
AE 17
AD 16
AT 130
AV 100
C : 164
CR 50
CG 13
CF 15
CS 24
CW 12
CY 50
D : 186
DG 36
DE 25
DH 22
DA 10
DY 16
DN 56
DV 21
E : 188
EG 29
ED 26
EA 4
EV 10
EK 101
EQ 18
F : 113
FL 38
FI 10
FC 13
FY 7
FV 11
FS 34
G : 385
GE 38
GD 52
GC 16
GA 18
GW 11
GV 35
GR 129
GS 86
H : 96
HQ 15
HP 10
HN 3
HL 4
HD 9
HR 30
HY 25
I : 158
IT 40
IS 8
IR 1
IN 10
IM 11
IL 14
IK 4
IF 6
IV 64
K : 104
KM 1
KI 1
KE 38
KN 16
KT 8
KQ 8
KR 32
L : 277
LP 117
LM 9
LI 7
LH 5
LF 39
LW 5
LV 34
LS 16
LR 33
LQ 12
M : 118
ML 6
MK 7
MI 28
MR 12
MT 30
MV 35
N : 122
NK 23
NI 5
NH 8
ND 14
NY 6
NT 2
NS 64
P : 262
PL 116
PH 11
PA 19
PT 19
PQ 10
PR 30
PS 57
Q : 108
QK 5
QL 6
QH 24
QP 17
QE 7
QR 49
R : 763
RH 129
RG 37
RC 151
RI 2
RK 16
RL 30
RM 3
RP 38
RQ 169
RW 153
RT 7
RS 28
S : 270
SI 11
SG 21
SF 29
SL 44
SC 16
SA 8
SY 7
SW 8
SN 31
ST 22
SP 50
SR 23
T : 247
TI 48
TA 56
TK 11
TS 19
TR 14
TM 61
TN 16
TP 22
V : 267
VG 15
VF 14
VE 7
VD 14
VA 46
VI 74
VL 31
VM 66
W : 67
WG 10
WC 15
WL 5
WS 6
WR 31
Y : 87
YC 49
YS 5
YN 3
YD 7
YF 6
YH 17

(d)

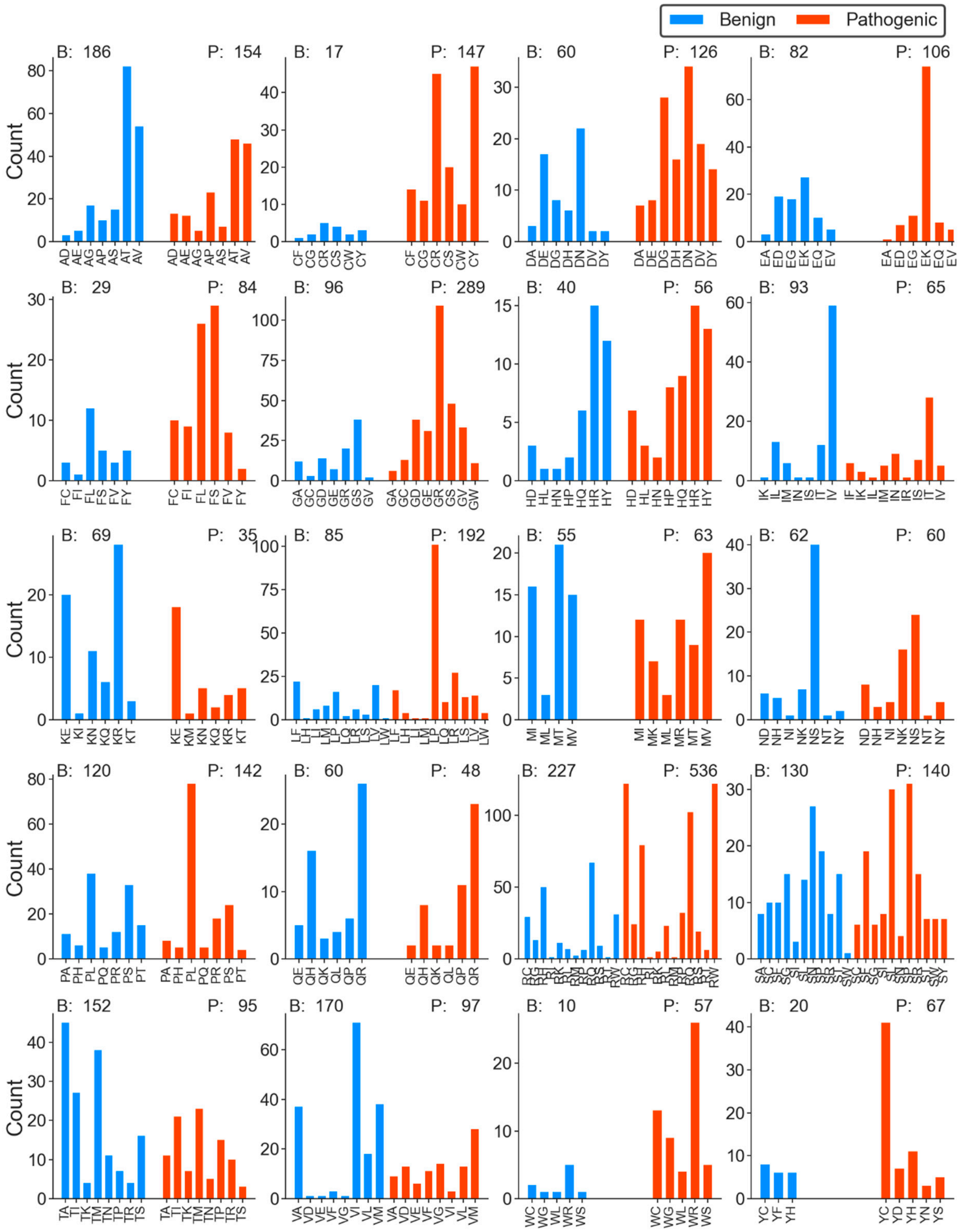

Benign
Pathogenic
Count
B: 186
P: 154
B: 17
P: 147
B: 60
P: 126
B: 82
P: 106
B: 29
P: 84
B: 96
P: 289
B: 40
P: 56
B: 93
P: 65
B: 69
P: 35
B: 85
P: 192
B: 55
P: 63
B: 62
P: 60
B: 120
P: 142
B: 60
P: 48
B: 227
P: 536
B: 130
P: 140
B: 152
P: 95
B: 170
P: 97
B: 10
P: 57
B: 20
P: 67

(e)

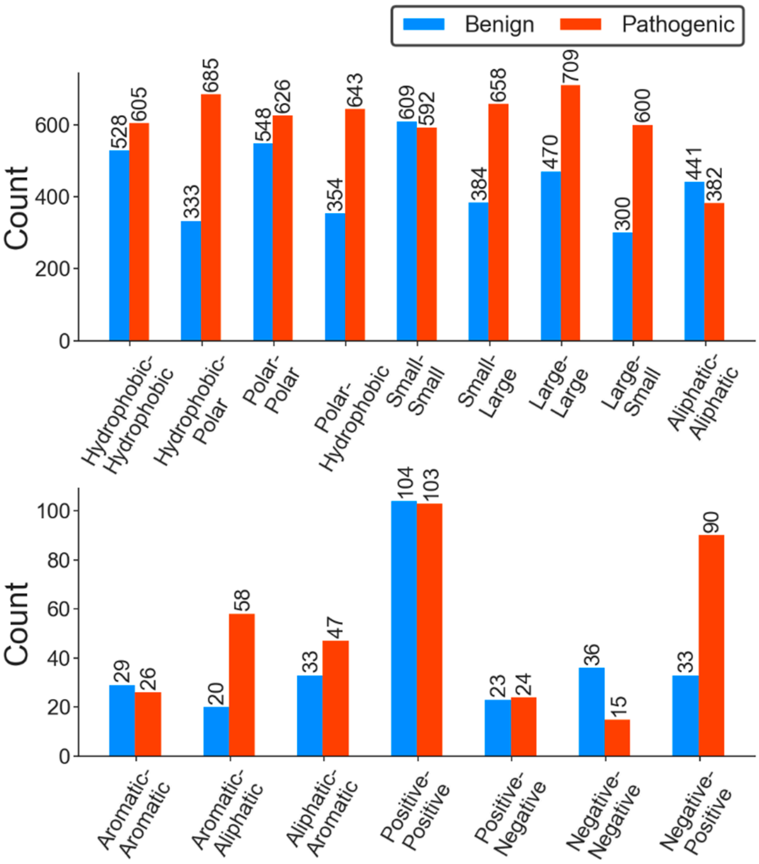

(f)

Figure S1. (**a**). Pie-chart summarising count of different amino acid mutations in the Monogenic Disorder Dataset 1 (no likely cases). In each subplot, the title shows the count of wild-type amino acids in the whole dataset, and the two-letter label of every pie-chart denotes the one-letter codes of the wild-type and mutant amino acid pair, respectively. (**b**). Bar plots summarising count of benign and pathogenic mutations in the Monogenic Disorder Dataset 1 (no likely cases). The two-letter label of every bar denotes the one-letter codes of the wild-type and mutant amino acid pairs, respectively. B denotes benign and P denotes pathogenic mutations. (**c**). Benign *vs.* pathogenic mutations assigned according to the property of amino acid mutation in the Monogenic Disorder Dataset 1 (no likely cases). The two-word label in the bar plot represents the chemical nature of the wild-type and mutant residues pairs, respectively. The amino acids according to their chemical properties are categorised as follows: Ala, Cys, Gly, Ile, Leu, Met, Phe, Pro, Trp, and Val are categorised as hydrophobic. Asp, Glu, Lys, Arg, His, Asn, Gln, Ser, Thr, and Tyr are categorised as polar. His, Phe, Trp, and Tyr as aromatic. Ala, ILe, Lys, Leu, Met, Pro, and Val as aliphatic. His, Lys, Arg as positive. Asp, and Glu as negative. Ala, Cys, Gly, Ser, Asn, Asp, Pro, Thr, and Val as small. And Arg, Gln, Glu, His, ILe, Leu, Lys, Met, Phe, Trp, and Tyr as large amino acids. (**d**). Pie-chart summarising count of different amino acid mutations in the Monogenic Disorder Dataset 2 (including likely cases). In each subplot, the title shows the count of wild-type amino acids in the whole dataset, and the two-letter label of every pie-chart denotes the one-letter codes of the wild-type and mutant amino acid pair, respectively. (**e**). Bar plots summarising count of benign and pathogenic mutations in the Monogenic Disorder Dataset 2 (including likely cases). The two-letter label of every bar denotes the one-letter codes of the wild-type and mutant amino acid pairs, respectively. B denotes benign and P denotes pathogenic mutations. (**f**). Benign *vs.* pathogenic mutations assigned according to the property of amino acid mutation in the Monogenic Disorder Dataset 2 (including likely cases). The two-word label in the bar plot represents the chemical nature of the wild-type and mutant residues pairs, respectively. The amino acids according to their chemical properties are categorised as follows: Ala, Cys, Gly, Ile, Leu, Met, Phe, Pro, Trp, and Val are categorised as hydrophobic. Asp, Glu, Lys, Arg, His, Asn, Gln, Ser, Thr, and Tyr are categorised as polar. His, Phe, Trp, and Tyr as aromatic. Ala, ILe, Lys, Leu, Met, Pro, and Val as aliphatic. His, Lys, Arg as positive. Asp, and Glu as negative. Ala, Cys, Gly, Ser,

Asn, Asp, Pro, Thr, and Val as small. And Arg, Gln, Glu, His, ILe, Leu, Lys, Met, Phe, Trp, and Tyr as large amino acids.

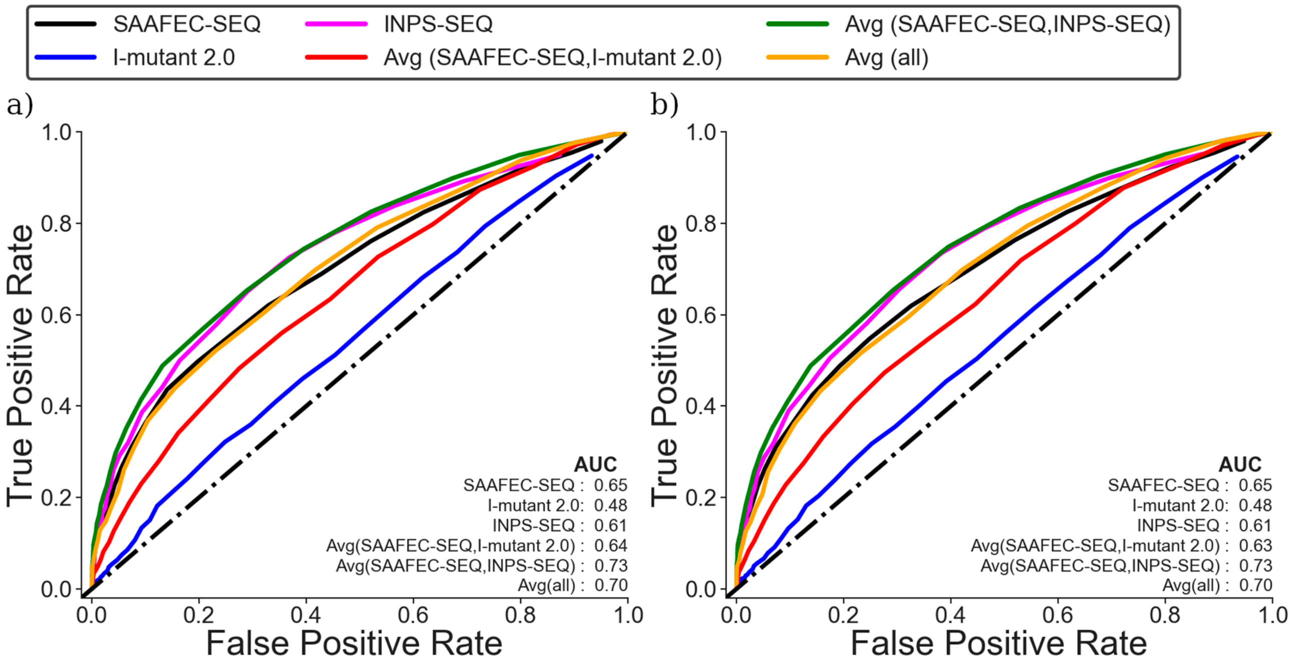

Figure S2. ROC curve for monogenic disorder dataset using absolute ΔΔ G values. (a) Monogenic Disorder Dataset 1 (no likely cases) (b) Monogenic Disorder Dataset 2 (including likely cases).

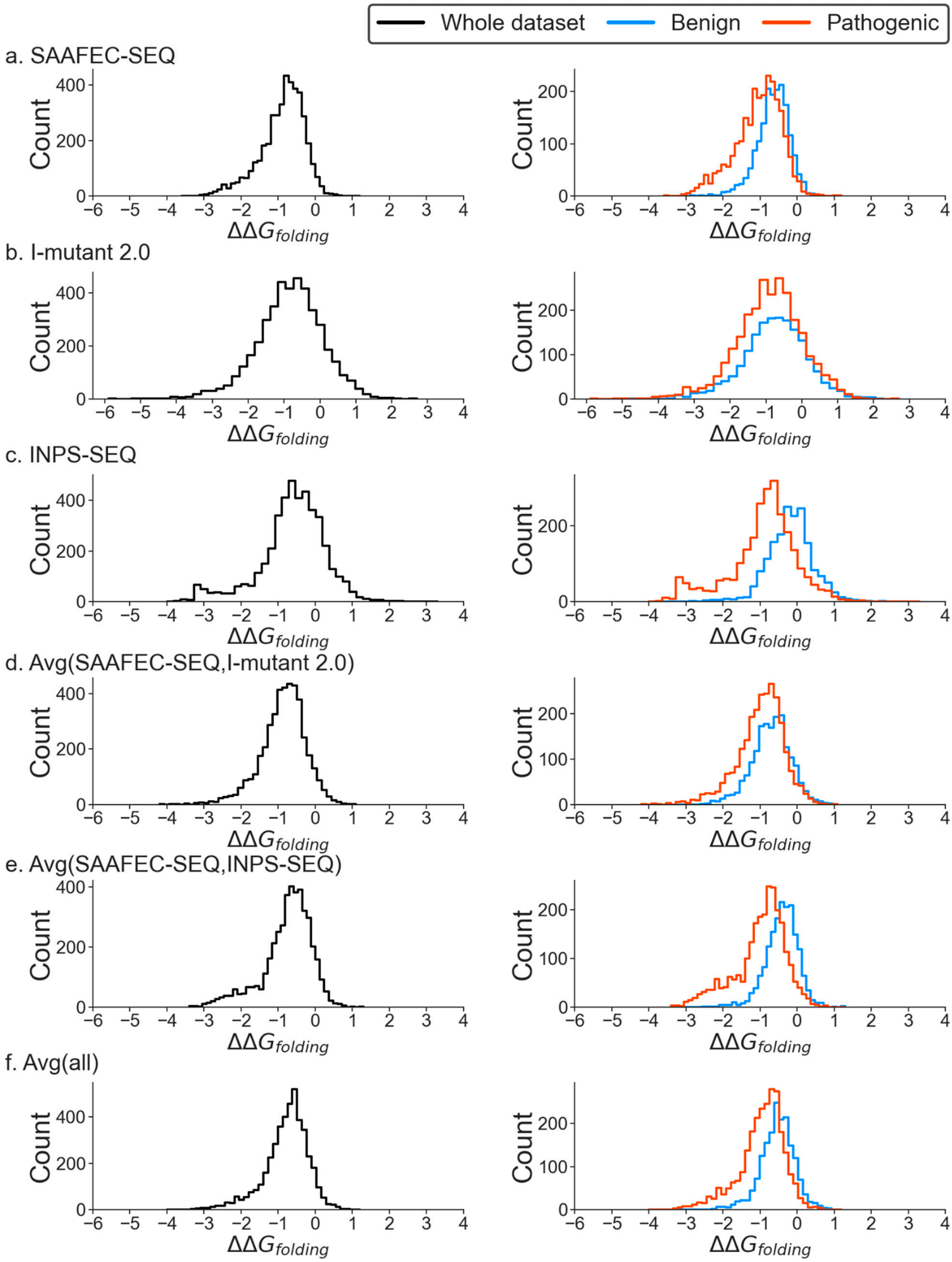

Figure S3. Distribution of change in folding free energy using different predictors using Monogenic Disorder Dataset 2 (including likely cases).

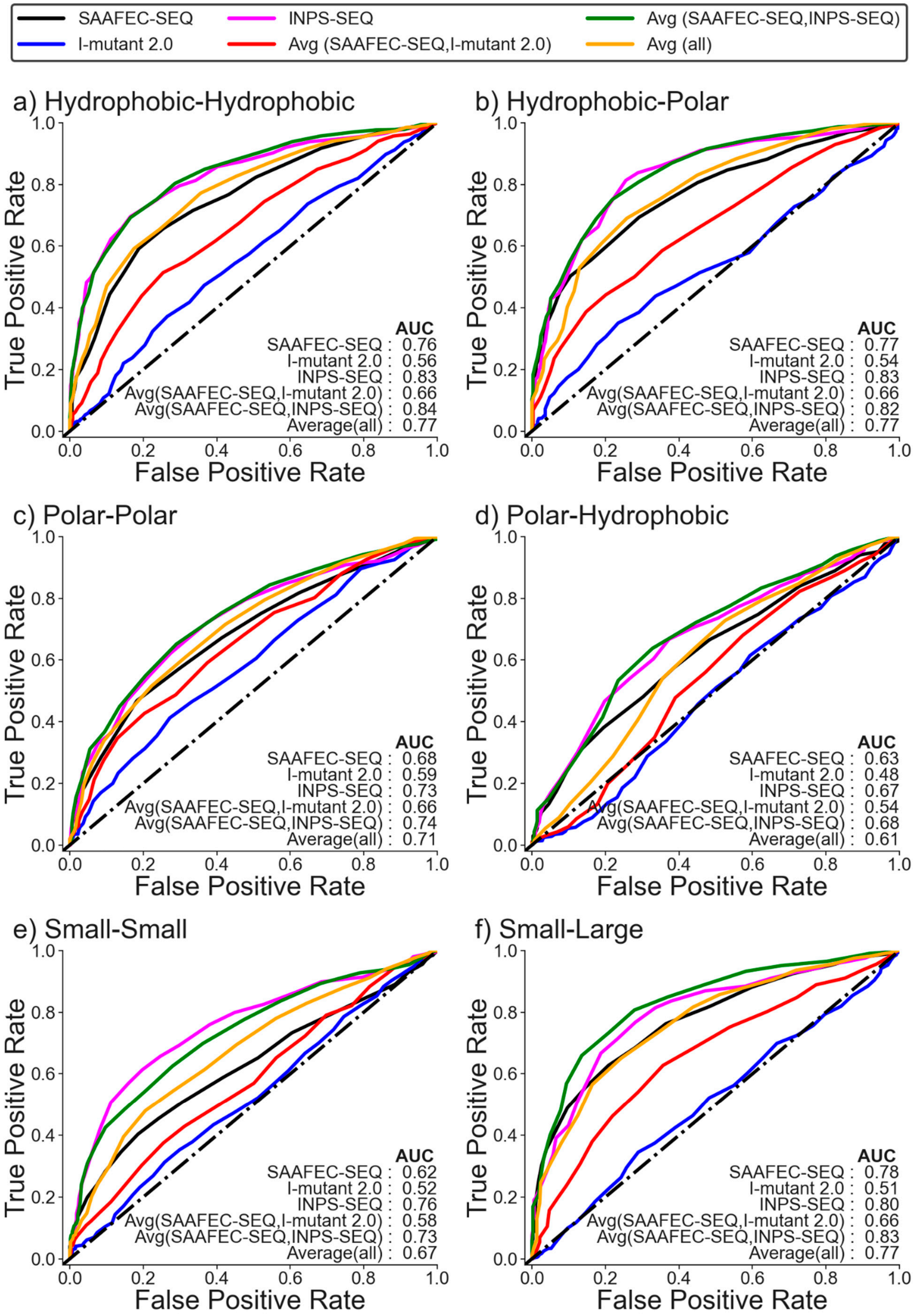
SAAFEC-SEQ
INPS-SEQ
Avg (SAAFEC-SEQ,INPS-SEQ)
I-mutant 2.0
Avg (SAAFEC-SEQ,I-mutant 2.0)
Avg (all)
a) Hydrophobic-Hydrophobic
AUC
SAAFEC-SEQ : 0.76
I-mutant 2.0 : 0.56
INPS-SEQ : 0.83
Avg(SAAFEC-SEQ,I-mutant 2.0) : 0.66
Avg(SAAFEC-SEQ,INPS-SEQ) : 0.84
Average(all) : 0.77
b) Hydrophobic-Polar
AUC
SAAFEC-SEQ : 0.77
I-mutant 2.0 : 0.54
INPS-SEQ : 0.83
Avg(SAAFEC-SEQ,I-mutant 2.0) : 0.66
Avg(SAAFEC-SEQ,INPS-SEQ) : 0.82
Average(all) : 0.77
c) Polar-Polar
AUC
SAAFEC-SEQ : 0.68
I-mutant 2.0 : 0.59
INPS-SEQ : 0.73
Avg(SAAFEC-SEQ,I-mutant 2.0) : 0.66
Avg(SAAFEC-SEQ,INPS-SEQ) : 0.74
Average(all) : 0.71
d) Polar-Hydrophobic
AUC
SAAFEC-SEQ : 0.63
I-mutant 2.0 : 0.48
INPS-SEQ : 0.67
Avg(SAAFEC-SEQ,I-mutant 2.0) : 0.54
Avg(SAAFEC-SEQ,INPS-SEQ) : 0.68
Average(all) : 0.61
e) Small-Small
AUC
SAAFEC-SEQ : 0.62
I-mutant 2.0 : 0.52
INPS-SEQ : 0.76
Avg(SAAFEC-SEQ,I-mutant 2.0) : 0.58
Avg(SAAFEC-SEQ,INPS-SEQ) : 0.73
Average(all) : 0.67
f) Small-Large
AUC
SAAFEC-SEQ : 0.78
I-mutant 2.0 : 0.51
INPS-SEQ : 0.80
Avg(SAAFEC-SEQ,I-mutant 2.0) : 0.66
Avg(SAAFEC-SEQ,INPS-SEQ) : 0.83
Average(all) : 0.77
True Positive Rate
False Positive Rate
0.0
0.2
0.4
0.6
0.8
1.0

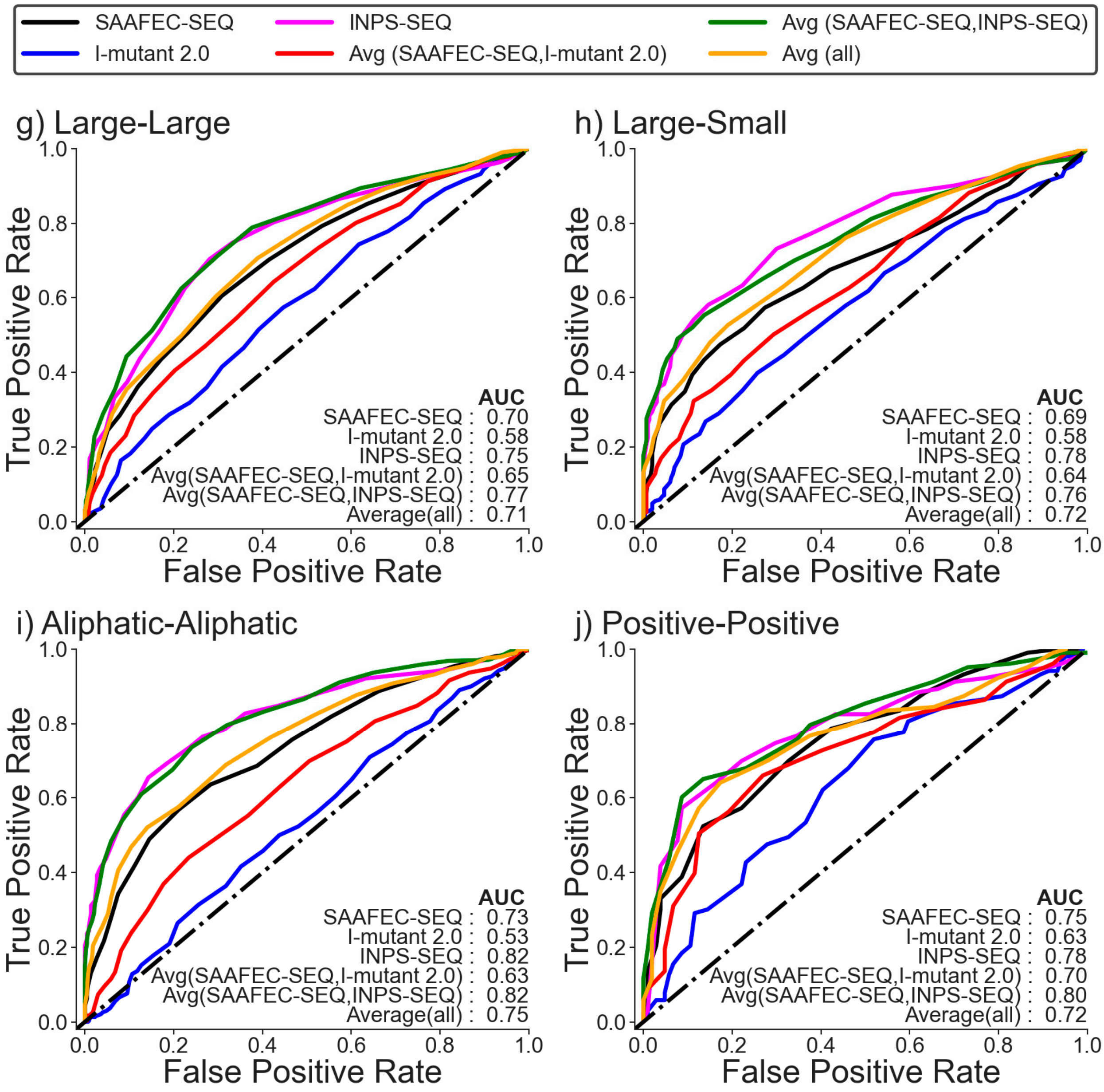

Figure S4. ROC curve for Monogenic Disorder Dataset 2 (including likely cases) for different categories of amino acid mutations. The amino acids according to their chemical properties are categorised as follows: Ala, Cys, Gly, Ile, Leu, Met, Phe, Pro, Trp, and Val are categorised as hydrophobic. Asp, Glu, Lys, Arg, His, Asn, Gln, Ser, Thr, and Tyr are categorised as polar. His, Phe, Trp, and Tyr as aromatic. Ala, ILe, Lys, Leu, Met, Pro, and Val as aliphatic. His, Lys, Arg as positive. Asp, and Glu as negative. Ala, Cys, Gly, Ser, Asn, Asp, Pro, Thr, and Val as small. And Arg, Gln, Glu, His, ILe, Leu, Lys, Met, Phe, Trp, and Tyr as large amino acids.

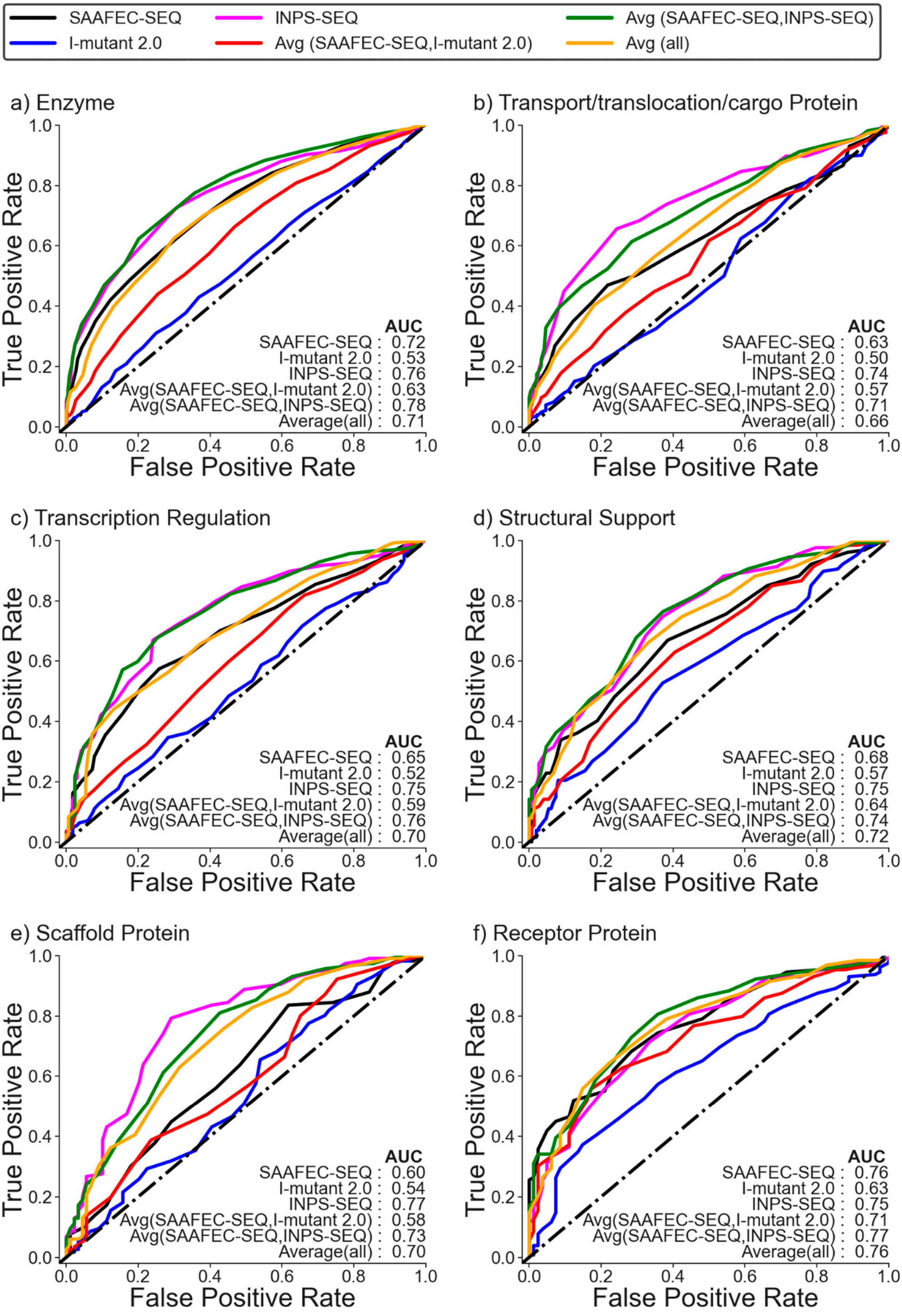
SAAFEC-SEQ
I-mutant 2.0
INPS-SEQ
Avg (SAAFEC-SEQ,I-mutant 2.0)
Avg (SAAFEC-SEQ,INPS-SEQ)
Avg (all)
a) Enzyme
b) Transport/translocation/cargo Protein
c) Transcription Regulation
d) Structural Support
e) Scaffold Protein
f) Receptor Protein
True Positive Rate
False Positive Rate
AUC
a) SAAFEC-SEQ : 0.72
I-mutant 2.0 : 0.53
INPS-SEQ : 0.76
Avg(SAAFEC-SEQ,I-mutant 2.0) : 0.63
Avg(SAAFEC-SEQ,INPS-SEQ) : 0.78
Average(all) : 0.71
b) SAAFEC-SEQ : 0.63
I-mutant 2.0 : 0.50
INPS-SEQ : 0.74
Avg(SAAFEC-SEQ,I-mutant 2.0) : 0.57
Avg(SAAFEC-SEQ,INPS-SEQ) : 0.71
Average(all) : 0.66
c) SAAFEC-SEQ : 0.65
I-mutant 2.0 : 0.52
INPS-SEQ : 0.75
Avg(SAAFEC-SEQ,I-mutant 2.0) : 0.59
Avg(SAAFEC-SEQ,INPS-SEQ) : 0.76
Average(all) : 0.70
d) SAAFEC-SEQ : 0.68
I-mutant 2.0 : 0.57
INPS-SEQ : 0.75
Avg(SAAFEC-SEQ,I-mutant 2.0) : 0.64
Avg(SAAFEC-SEQ,INPS-SEQ) : 0.74
Average(all) : 0.72
e) SAAFEC-SEQ : 0.60
I-mutant 2.0 : 0.54
INPS-SEQ : 0.77
Avg(SAAFEC-SEQ,I-mutant 2.0) : 0.58
Avg(SAAFEC-SEQ,INPS-SEQ) : 0.73
Average(all) : 0.70
f) SAAFEC-SEQ : 0.76
I-mutant 2.0 : 0.63
INPS-SEQ : 0.75
Avg(SAAFEC-SEQ,I-mutant 2.0) : 0.71
Avg(SAAFEC-SEQ,INPS-SEQ) : 0.77
Average(all) : 0.76

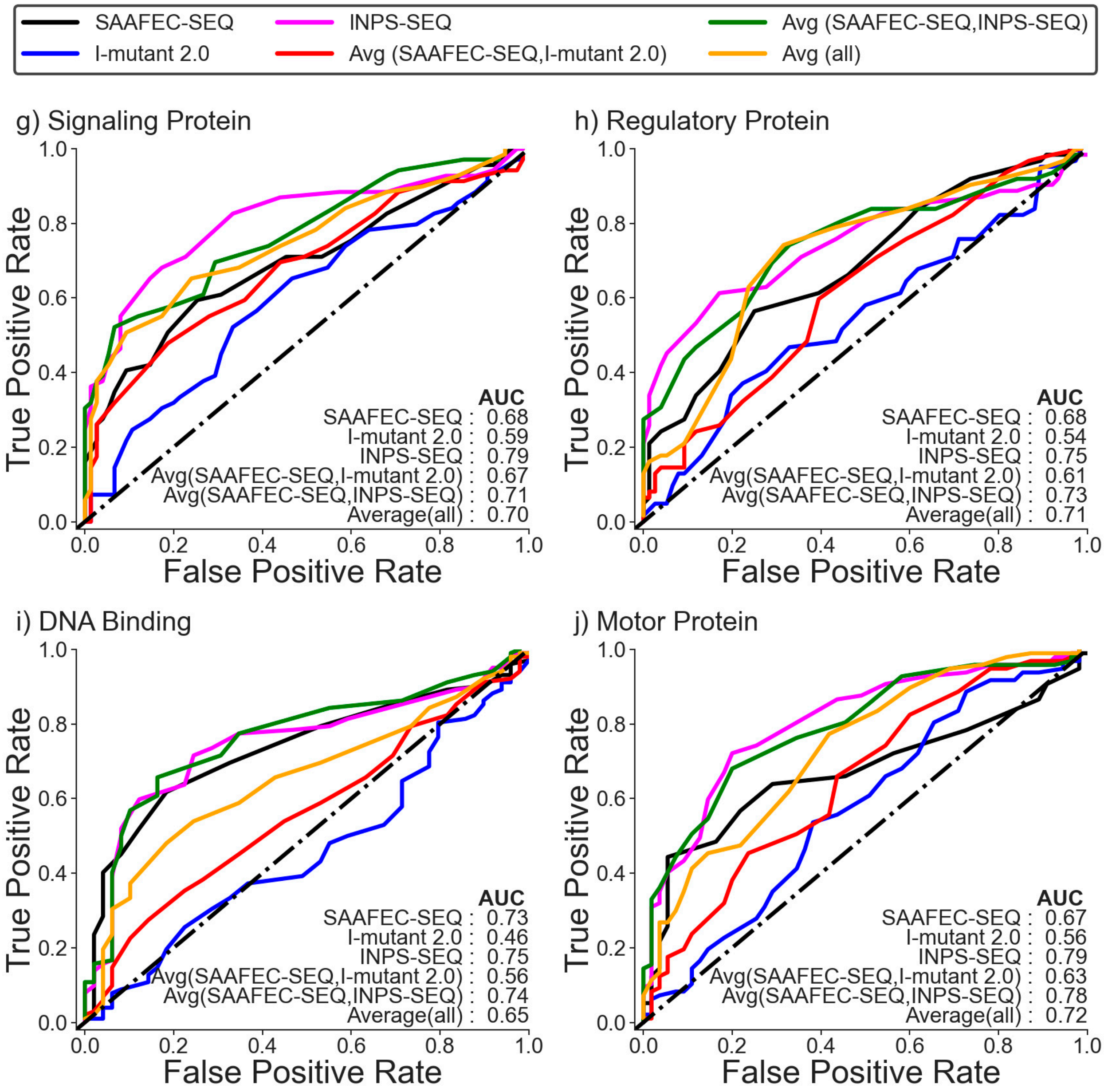

Figure S5. ROC curve for Monogenic Disorder Dataset 2 (including likely cases) for different functional categories.

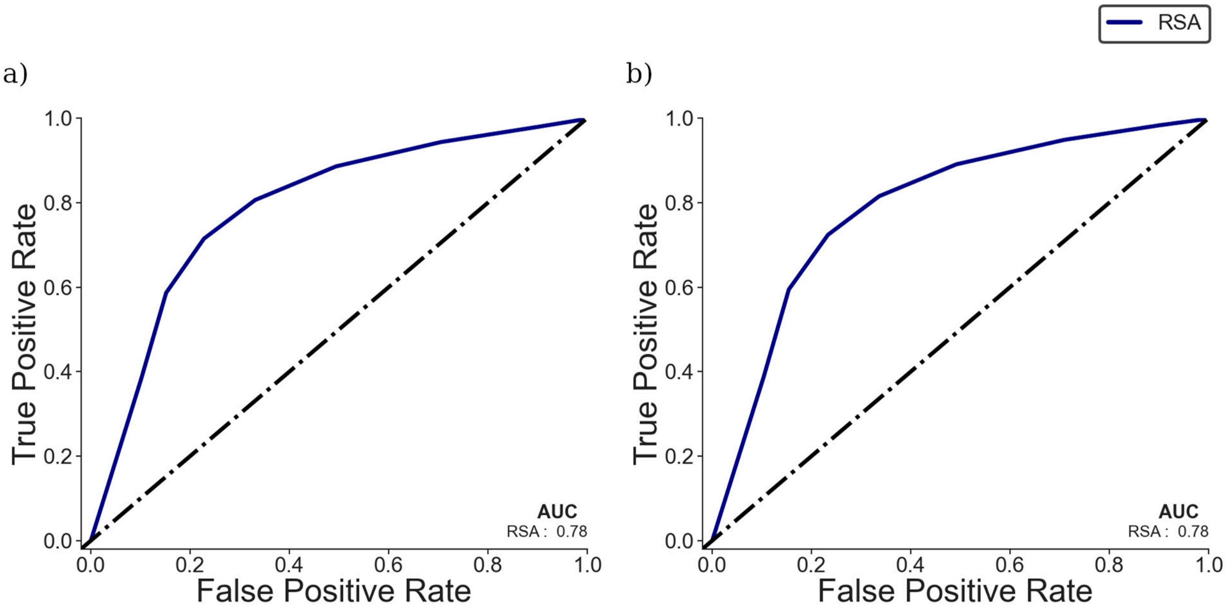

Figure S6. ROC curve for monogenic disorder dataset using Relative Surface Area (RSA) of amino acid. (a) Monogenic Disorder Dataset 1 (no likely cases) (b) Monogenic Disorder Dataset 2 (including likely cases).

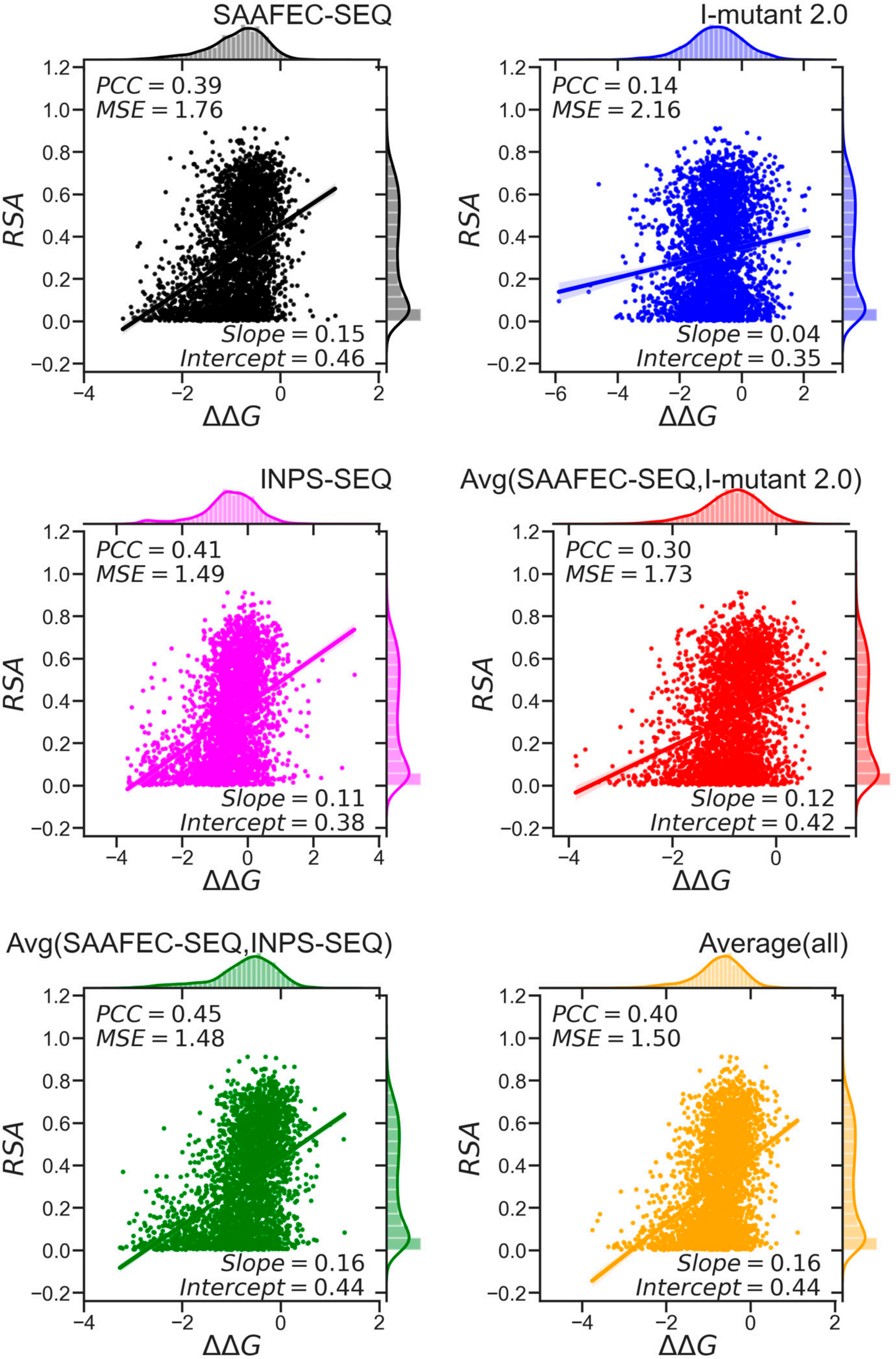
SAAFEC-SEQ
PCC = 0.39
MSE = 1.76
Slope = 0.15
Intercept = 0.46
I-mutant 2.0
PCC = 0.14
MSE = 2.16
Slope = 0.04
Intercept = 0.35
INPS-SEQ
PCC = 0.41
MSE = 1.49
Slope = 0.11
Intercept = 0.38
Avg(SAAFEC-SEQ,I-mutant 2.0)
PCC = 0.30
MSE = 1.73
Slope = 0.12
Intercept = 0.42
Avg(SAAFEC-SEQ,INPS-SEQ)
PCC = 0.45
MSE = 1.48
Slope = 0.16
Intercept = 0.44
Average(all)
PCC = 0.40
MSE = 1.50
Slope = 0.16
Intercept = 0.44
RSA
ΔΔG

(a)

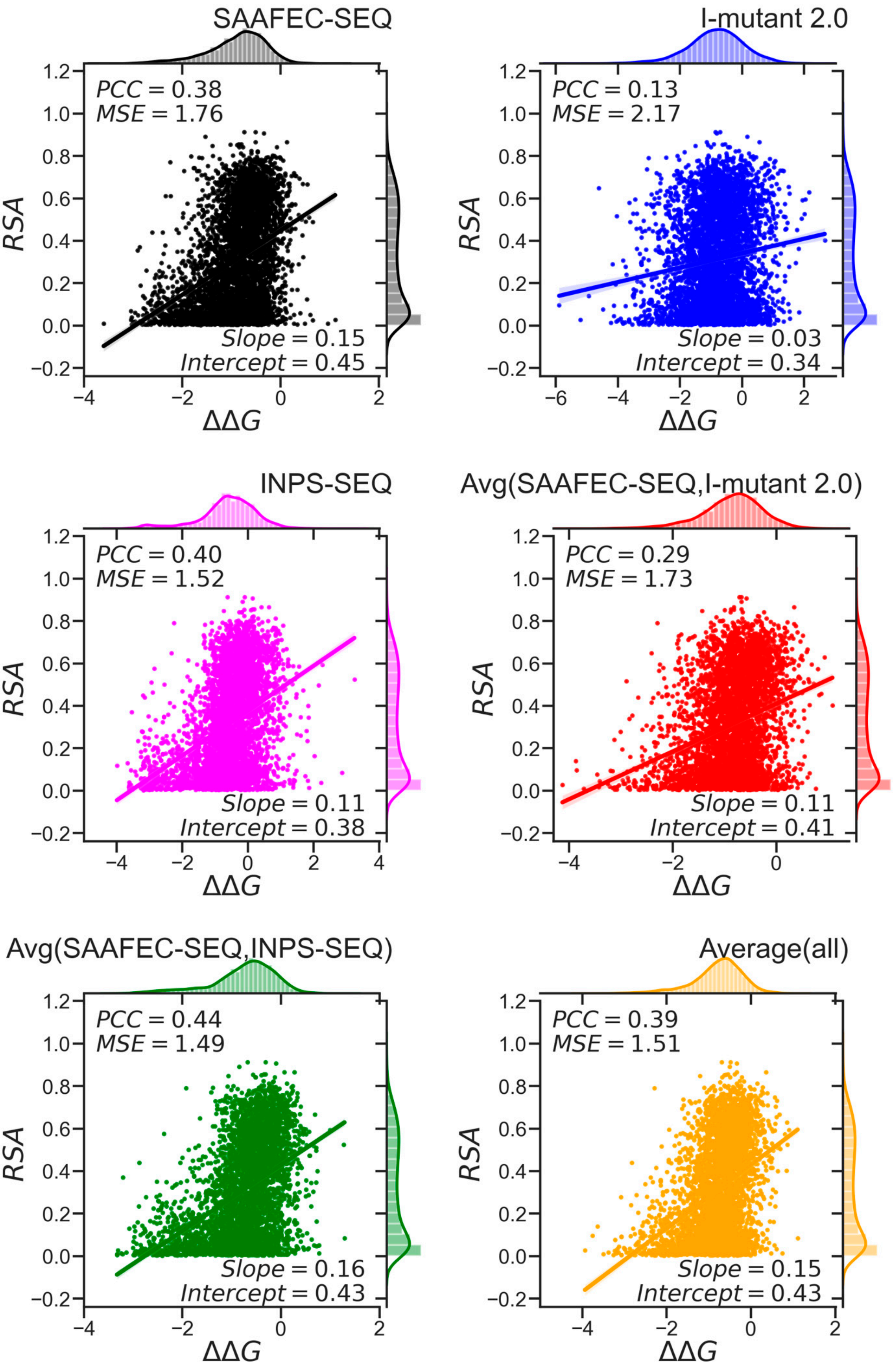
SAAFEC-SEQ
PCC = 0.38
MSE = 1.76
Slope = 0.15
Intercept = 0.45
I-mutant 2.0
PCC = 0.13
MSE = 2.17
Slope = 0.03
Intercept = 0.34
INPS-SEQ
PCC = 0.40
MSE = 1.52
Slope = 0.11
Intercept = 0.38
Avg(SAAFEC-SEQ,I-mutant 2.0)
PCC = 0.29
MSE = 1.73
Slope = 0.11
Intercept = 0.41
Avg(SAAFEC-SEQ,INPS-SEQ)
PCC = 0.44
MSE = 1.49
Slope = 0.16
Intercept = 0.43
Average(all)
PCC = 0.39
MSE = 1.51
Slope = 0.15
Intercept = 0.43
RSA
ΔΔG

(b)

Figure S7. (**a**). Plots of $\Delta\Delta G$ against RSA for Monogenic Disorder Dataset 1 (no likely cases). (**b**). Plots of $\Delta\Delta G$ against RSA for Monogenic Disorder Dataset 2 (including likely cases).

Supplementary Tables

Table S1. Total number of amino acid mutations based on chemical nature of amino acid mutations in monogenic disorder database.

Se. No.	Type of amino acid mutation	Dataset 1			Dataset 2		
		Benign	Pathogenic	Total	Benign	Pathogenic	Total
1.	Hydrophobic-Hydrophobic	424	460	884	528	605	1133
2.	Hydrophobic-Polar	268	529	797	333	685	1018
3.	Polar-Polar	436	465	901	548	626	1174
4.	Polar-Hydrophobic	277	480	757	354	643	997
5.	Small-Small	478	463	941	609	592	1201
6.	Small-Large	307	494	801	384	658	1042
7.	Large-Large	370	514	884	470	709	1179
8.	Large-Small	250	463	713	300	600	900
9.	Aliphatic-Aliphatic	352	301	653	441	382	823
10.	Aliphatic-Aromatic	25	34	59	33	47	80
11.	Aromatic-Aromatic	24	18	42	29	26	55
12.	Aromatic-Aliphatic	17	36	53	20	58	78
13.	Positive-Positive	88	69	157	104	103	207
14.	Positive-Negative	19	21	40	23	24	47
15.	Negative-Negative	26	15	41	36	15	51
16.	Negative-Positive	23	67	90	33	90	123

Table S2. Total number of amino acid mutations based on functional annotation of proteins in monogenic disorder database.

Se. No.	Functional Class	No. of entries in Dataset 1	No. of entries in Dataset 2
1.	Enzyme	1295	1646
2.	Transport/translocation/cargo Protein	445	570
3.	Transcription Regulation	343	420
4.	Structural Support	224	275
5.	Scaffold Protein	152	205
6.	Receptor Protein	141	210
7.	Signalling Protein	128	144
8.	Regulatory Protein	112	138
9.	DNA Binding	90	151
10.	Motor Protein	87	152
11.	Secretory Proteins	74	80
12.	Adhesion Protein	66	87
13.	Chaperons	66	74
14.	Membrane Protein	32	40
15.	RNA Binding	31	50
16.	Antigen-Antibody	14	18
17.	Miscellaneous	39	62
